# Cueing-assisted gamified augmented-reality home rehabilitation for gait and balance in people with Parkinson disease: feasibility and effectiveness in the clinical pathway

**DOI:** 10.1093/ptj/pzag012

**Published:** 2026-02-10

**Authors:** Eva M Hoogendoorn, Daphne J Geerse, Annejet T van Dam, Sybren J van Hall, Pieter F van Doorn, Lotte E S Hardeman, Marco J M Hoozemans, John F Stins, Melvyn Roerdink

**Affiliations:** Department of Human Movement Sciences, Faculty of Behavioural and Movement Sciences, Vrije Universiteit Amsterdam, Amsterdam Movement Sciences, Amsterdam, The Netherlands; Department of Nutrition and Movement Sciences, NUTRIM Institute of Nutrition and Translational Research in Metabolism and MHeNs Institute of Mental Health and Neurosciences, Faculty of Health, Medicine and Life Sciences, Maastricht University, Maastricht, The Netherlands; Department of Human Movement Sciences, Faculty of Behavioural and Movement Sciences, Vrije Universiteit Amsterdam, Amsterdam Movement Sciences, Amsterdam, The Netherlands; Department of Human Movement Sciences, Faculty of Behavioural and Movement Sciences, Vrije Universiteit Amsterdam, Amsterdam Movement Sciences, Amsterdam, The Netherlands; Department of Human Movement Sciences, Faculty of Behavioural and Movement Sciences, Vrije Universiteit Amsterdam, Amsterdam Movement Sciences, Amsterdam, The Netherlands; Department of Human Movement Sciences, Faculty of Behavioural and Movement Sciences, Vrije Universiteit Amsterdam, Amsterdam Movement Sciences, Amsterdam, The Netherlands; Department of Nutrition and Movement Sciences, NUTRIM Institute of Nutrition and Translational Research in Metabolism and MHeNs Institute of Mental Health and Neurosciences, Faculty of Health, Medicine and Life Sciences, Maastricht University, Maastricht, The Netherlands; Department of Human Movement Sciences, Faculty of Behavioural and Movement Sciences, Vrije Universiteit Amsterdam, Amsterdam Movement Sciences, Amsterdam, The Netherlands; Department of Human Movement Sciences, Faculty of Behavioural and Movement Sciences, Vrije Universiteit Amsterdam, Amsterdam Movement Sciences, Amsterdam, The Netherlands; Department of Human Movement Sciences, Faculty of Behavioural and Movement Sciences, Vrije Universiteit Amsterdam, Amsterdam Movement Sciences, Amsterdam, The Netherlands; Department of Human Movement Sciences, Faculty of Behavioural and Movement Sciences, Vrije Universiteit Amsterdam, Amsterdam Movement Sciences, Amsterdam, The Netherlands; Department of Nutrition and Movement Sciences, NUTRIM Institute of Nutrition and Translational Research in Metabolism and MHeNs Institute of Mental Health and Neurosciences, Faculty of Health, Medicine and Life Sciences, Maastricht University, Maastricht, The Netherlands; Department of Science, Strolll Ltd., Stafford ST16 2LP, UK

**Keywords:** Parkinson disease, augmented reality, cueing, gait, balance, rehabilitation, gamified exercise, clinical practice, telerehabilitation, blended care

## Abstract

**Importance:**

Physical therapy is moving toward digitally supported, independent, home-based care to improve therapy accessibility and adherence.

**Objective:**

This trial evaluated the clinical feasibility and potential effectiveness of Strolll, an augmented reality (AR) neurorehabilitation platform offering gamified gait-and-balance exercises with optional assistive AR cueing for individuals with Parkinson disease, implemented in real-world clinical practice.

**Design and setting:**

In this pragmatic clinical trial, 15 Dutch health care practices were onboarded, 28 therapists trained, and 100 individuals with Parkinson disease (Hoehn and Yahr stages 1-3) included. All participants followed the T0-usual-care-control-T1-Strolll-intervention-T2 procedure.

**Intervention:**

The Strolll intervention consisted of 2-week supervised in-clinic training followed by 6 weeks, 5 sessions per week of 30 active minutes each, independent home-based training.

**Results:**

No serious adverse events occurred; only 2 non-injurious falls were reported in >60.000 exercise minutes. Adherence was high (96% session adherence, 91% active minutes/session adherence). Therapists prescribed the program progressively, with significantly higher game-play levels over time. Participants’ exercise performance increased over time. Participants and therapists rated user experience and technology acceptance positively. Timed “Up & Go” test and 10-meter walk test (10MWT) (fast speed) scores improved significantly after the intervention period only. Five times sit-to-stand test, 10MWT (comfortable speed), and Mini Balance Evaluation Systems Test scores improved after both usual-care and intervention periods. Falls Efficacy Scale-International scores showed no significant improvements. AR cueing was deemed beneficial for a subset of participants.

**Conclusions:**

Strolll is a safe, adherable, progressive, usable, and well-accepted therapist-managed, home-based intervention for people with Parkinson disease, with the potential to improve gait, balance, and fall-risk indicators. Findings on the integration of AR cueing highlight the importance of an individualized approach.

**Relevance:**

Implementing AR rehabilitation technologies like Strolll in the clinical pathway is feasible, offering a safe and scalable way for individuals to train independently, potentially improving accessibility of care and broadening its use to physical activity promotion.

**Clinical trial registration:**

This study was registered at ClinicalTrials.gov (NCT06590987).

## Introduction

Parkinson disease is the fastest-growing, progressive neurological disease with a severe impact on the health care system.^1^ Gait-and-balance impairments are common in people with Parkinson disease, contributing to an increased risk of falls and a reduced quality of life.^2^ Exercise and physical therapy are considered essential for people with Parkinson disease to maintain and improve motor function, decelerate further decline, and promote overall health and well-being.^3^^,^^4^ Nevertheless, due to mobility impairments and other practical limitations, individuals with Parkinson disease often face challenges attending to regular, center-based rehabilitation programs over a prolonged period,^5^ while adherence rates to prescribed exercise at home are typically low.^6^ Various home-based rehabilitation interventions have emerged for maintaining and enhancing motor function and achieving overall health benefits.^7^^,^^8^ Despite their potential, traditional home-based exercise interventions often have low therapy adherence and limited monitoring capabilities for therapists.^9^ Emerging technologies with gamified exercises and captured exercise data offer promising and engaging solutions to these limitations.^10^

**Figure 1:**
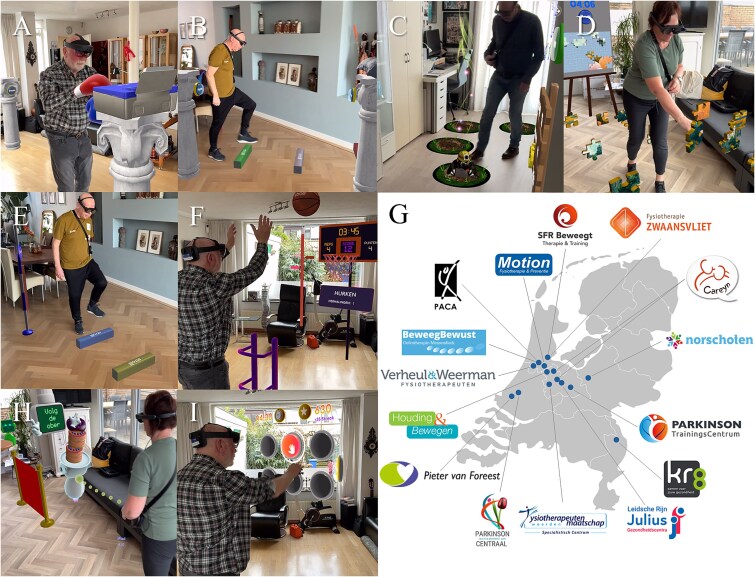
Strolll gait-and-balance exercises (A-F and H-I) implemented in the clinical pathway in the Netherlands (G). Specific exercises were Smash! (without [A] and with [B] assistive AR cues), Mole Patrolll (C), Puzzle Walk (D), Cue Challenge (E), Basketballl (F), Wobbly Waiter (H), and Hot Buttons (I).

Augmented reality (AR) glasses, such as Magic Leap 2 or Microsoft HoloLens 2, represent a promising innovation in this domain, allowing for an interactive experience within an individual’s environment. This technology is embodied in the development of Strolll, an AR neurorehabilitation platform with gamified gait-and-balance exercises specifically designed for people with Parkinson disease for use in the clinic or at home (see Figure 1A-F and H-I, Video S1), which can be managed by therapists for supervised in-clinic therapy as well as for asynchronous telerehabilitation independently at home.^11^ A recent controlled feasibility study in a science-led setting demonstrated that the earlier version of Strolll (previously called Reality DTx) is safe, usable, adherable, and well-accepted for independent use at home, with targeted intervention effects for improving gait, balance, and fall risk in people with Parkinson disease.^12^ A key innovation of the Strolll platform is the integration of AR cueing, involving visual or auditory stimuli to assist and modify gait during the gamified exercises.^13^ These AR cues may be used by individuals with more severe gait impairments, like freezing of gait and festination, to assist them with AR exercises that require walking and turning. Since cueing is not a one-size-fits-all solution,^14–16^ these AR cues are probably most beneficial when tailored to the individual (ie, type of cue and settings).

This pragmatic clinical trial is designed to investigate the clinical feasibility and potential effectiveness of Strolll implemented in real-world clinical practice. We expected that Strolll is (1) clinically feasible in terms of safety, adherence, performance, user experience, and acceptability (for individuals with Parkinson disease and their therapists) and (2) potentially effective for improving gait, balance, and fall-risk indicators. Additionally, we evaluated procedures to (1) identify participants deemed to benefit from complementary AR cues in AR exercises and (2) individually tailor AR cue characteristics. We expected that a subset of participants, particularly those with severe mobility impairments, such as freezing of gait and festination, could benefit from adding complementary AR cues and that the selected type of cue and its settings varied over participants (ie, cueing is not a one-size-fits-all solution^15^^,^^16^).

## Methods

The trial was registered on Clinicaltrials.gov (NCT06590987). The methods are concisely described here, with a fully detailed study protocol previously published.^17^ Deviations from the published study protocol (ie, required adjustment of the statistical analysis^18^) are documented and justified in the Transparent Changes document (Suppl. Material 1).

### Study setting, design, and procedures

This study was implemented in the clinical pathway at 15 health care practices affiliated with ParkinsonNet, a Dutch specialized network for certified Parkinson’s therapists,^19^ across the Netherlands (Figure 1G): Pieter van Foreest, Houding & Bewegen, Verheul & Weerman Fysiotherapeuten, BeweegBewust, PACA, Motion Fysiotherapie & Preventie, SFR Beweegt, Fysiotherapie Zwaansvliet, Careyn, Norschoten, Parkinson TrainingsCentrum, Kr8, Leidsche Rijn Julius Gezondheidscentra, Fysiotherapeuten Maatschap Woerden, and Parkinson Centraal. All therapists involved (*n* = 28) were trained in using Strolll and familiarized with the study procedure, including administering the clinical assessments.^17^

This pragmatic clinical trial included 3 in-clinic assessments performed by the participant’s therapist, which we aimed to schedule at the same time of day to minimize the influence of medication and ON/OFF motor fluctuations. After the baseline assessment (T0), participants underwent a 6-week usual-care period, followed by a preintervention assessment (T1). During the usual-care period, participants received no additional instructions or therapy related to Strolll and continued their regular weekly therapy schedule, generally consisting of in-clinic sessions adhering to physical therapy guidelines for Parkinson disease,^3^^,^^4^ possibly supplemented by prescribed conventional home exercises (eg, paper-listed or video clips demonstrating the exercises). This was followed by 2 weeks of supervised in-clinic training with Strolll, after which participants used Strolll independently at home for 6 weeks, asynchronously managed by their therapists, and integrated with their regular in-clinic usual-care sessions. Participants finished the study with a postintervention assessment (T2). This design, where all participants followed the same T0-control-T1-intervention-T2 procedure, streamlined implementation in a real-world clinical setting and facilitated the assessment of both clinical feasibility and potential effectiveness.

### Study population

Participants (*n* = 100, based on an a-priori power analysis^17^) were recruited by their physical therapist or exercise therapist in the participating health care practices. Eligibility was established through telephone screening by the researchers. Participants were included if they had been diagnosed with Parkinson disease (stages 1-3 on the Hoehn and Yahr scale), experienced self-reported gait and/or balance impairments, were aged 21 years or over, and proficient in the Dutch language. Exclusion criteria included neurological and/or orthopedic conditions seriously affecting gait, severe cognitive, visual, and/or hearing impairments (after corrective aids), insufficient physical capacity (eg, frequent faller and/or the inability to walk independently for 30 min), severe visual hallucinations and/or illusions, and unstable dosage of medication at the start of the study. All participants provided written informed consent before participating in the study.

### Intervention

The Strolll intervention, a certified neurorehabilitation software platform developed for AR glasses in collaboration with Strolll Limited (www.strolll.co), was delivered in 2 stages. In the first stage, therapists introduced participants to Strolll during a 2-week supervised in-clinic training period, allowing participants to familiarize themselves with the technology and evaluate the potential benefits of AR cueing. Strolll comprises 7 complementary gamified exercises: Mole Patrolll, Smash!, Basketballl, Hot Buttons, Puzzle Walk, Wobbly Waiter, and Cue challenge, delivered via state-of-the-art AR glasses (HoloLens 2; Microsoft, and Magic Leap 2; Magic Leap, Inc.), see Figure 1A-F and H-I and Video S1, each designed to improve aspects of gait, balance, and fall risk. AR cues could be added to Smash! and Cue challenge (Video S2) to support or improve the participant’s gait (eg, increasing step length and alleviating freezing). During the in-clinic training period, all types of cues were explored (Suppl. Material 2) and tailored to the participant (eg, intercue distance, cue height, and color). Therapists were informed by baseline gait characteristics of participants (eg, step length) and observed in real time how participants responded to different cues. When deemed beneficial, the preferred AR cue was selected through a shared decision-making process involving the participant and therapist during the in-clinic training period and added to Smash! and Cue challenge. The other walking-based exercises, Mole Patrolll, Puzzle Walk, and Wobbly Waiter, already incorporated AR-mediated goal-directed elements acting as spatial location cues (moles, puzzle pieces) or spatiotemporal cues (moving waiter). A detailed description of the gamified exercises and cues can be found in Supplementary Material 2. At the end of the in-clinic period, therapists, in consultation with the participant, decided whether the Strolll intervention at home was deemed safe and suitable for the participants; if not, participants discontinued the study. The second stage comprised 6 weeks of training with Strolll independently at home, consisting of 30-minute sessions 5 days per week by default, following the recommended 150 exercise minutes per week,^20^ with adjustments based on individual capacity as determined by the therapist. The therapists were instructed to remotely prescribe, evaluate, and adjust the exercise program weekly, with the option to modify each gamified exercise separately in terms of duration, level, and settings, through the Strolll web portal. By default, all exercises were prescribed for each participant, although therapists could adjust the selection if needed. This process was guided by shared decision-making between participant and therapist using participant’s feedback and therapist’s clinical expertise, and potentially driven by adherence and game scores (eg, number of moles spawned, meters walked; see Suppl. Material 2) from the Strolll web portal as input.

### Outcomes

The clinical feasibility of Strolll was assessed in terms of safety (ie, adverse events while using Strolll, such as falls or dizziness, and weekly fall rate in daily life), adherence (ie, adherence to the prescribed number of sessions and the prescribed active minutes/session), performance (ie, game-related and functional performance scores), user experience, and acceptability. User experience and acceptability were evaluated using the User Experience Questionnaire (UEQ),^21^^,^^22^ a technology acceptance and use questionnaire (based on the model of Unified Theory of Acceptance and Use of Technology^23^), a top 3 advantages and disadvantages of Strolll for future clinical implementation, and a Strolll intervention-specific questionnaire including items on the integration of AR cues. Additionally, therapists’ experiences were assessed with comparable questionnaires, followed by a poststudy process evaluation in focus groups, which will be reported in a separate qualitative paper. A final feasibility outcome was the proportion of participants deemed eligible for training independently at home and the number of intervention-related dropouts.

Standardized clinical gait-and-balance assessments, which are indicators of fall risk,^24–27^ were administered to evaluate the potential effectiveness of the intervention at baseline, preintervention, and postintervention (T0, T1, and T2). The Timed “Up & Go” test (TUG) was the primary outcome used for the sample-size calculation, with as secondary outcomes the 5 times sit-to-stand test (5TSTS), 10-meter walk test (10MWT) at comfortable and fast speeds, Mini Balance Evaluation Systems Test (Mini-BESTest), and Falls Efficacy Scale-International (FES-I).

To evaluate the integration of AR cues within the gamified exercises, we documented whether participants used AR cues during the Strolll intervention, along with the specific type of AR cue, its settings (eg, length, height, and width), and the rationale for using the AR cue (eg, alleviating freezing of gait or increasing step length).

### Statistical analysis

Safety was described by the number of adverse events, including a paired-samples *t*-test comparison of the average weekly number of falls experienced during the usual-care and intervention periods. Adherence to the Strolll intervention was assessed with a repeated-measures ANOVA with the within-subject factor time (3 levels: first, second, and third part, so approximately 2-week intervals, of Strolll intervention at home). In case of significant main effects of time, post hoc paired-samples *t*-tests were conducted, with Bonferroni-adjusted *P*-values. With one-sample *t*-tests against 100%, we evaluated possible systematic deviations in adherence from what was prescribed. To assess whether Strolll integrated into the clinical pathway was prescribed in a progressive yet achievable manner, we subjected game levels and performance scores (see Suppl. Material 2) to repeated-measures ANOVAs or their non-parametric equivalents with time as a within-subject factor (3 levels: first, second, and third part), using paired-samples *t*-tests (or non-parametric equivalents) for post hoc analyses of significant main effects, with *P-*values adjusted using the Bonferroni correction. Since analyses were conducted independently for each gamified exercise, we did not apply an additional correction for multiple exercises beyond the within-analysis adjustments. The UEQ outcomes were described descriptively and interpreted against known benchmark scores.^22^ All other user experience and acceptability outcomes were processed descriptively, for both participants and therapists.

The potential effectiveness of Strolll on clinical gait-and-balance test outcomes was evaluated using a linear mixed-effects (LME) model with Time (T0, T1, and T2) as a fixed effect and participant as a random intercept. Contrast testing with the first (T1-T0) and second (T2-mean [T1, T0]) reverse Helmert contrasts was done to evaluate potential control and intervention effects, respectively. For transparency, we note that this effectiveness analysis deviated from the previously published study protocol,^17^ as justified in the transparent changes document (Suppl. Material 1). Results of the preplanned repeated-measures ANOVAs (within-subjects comparisons) were comparable with the results of the LME analyses (as detailed in Suppl. Material 3).

All statistical analyses were performed in JASP^28^ with significance set at α = .05. Missing data of feasibility outcomes were excluded analysis-by-analysis. For 3 participants, 1 or 2 missing items on the Mini-BESTest were imputed. For all repeated-measures ANOVAs, the Huynh–Feldt correction was applied if the Greenhouse–Geisser epsilon exceeded 0.750, otherwise, the Greenhouse–Geisser correction was used. Effect sizes were quantified as *η_ρ_*^2^.

### Role of the funding source

The funder played no role in the design, conduct, and reporting of this study.

## Results

### Participants

Participants were recruited between May and October 2024. Out of 132 recruited people with Parkinson disease assessed for eligibility, 100 enrolled in the study. These 100 participants had a mean (SD) age of 69.6 (8.2) years (range 42-87; 72 male, 28 female) and Hoehn and Yahr stages 1 (*n* = 26), 2 (*n* = 53), and 3 (*n* = 21), with 37 participants qualifying as freezers (defined by a non-zero score on the New Freezing Of Gait Questionnaire^29^). A detailed overview of participant flow and characteristics is provided in Supplementary Materials 4 and 5, respectively. After the supervised in-clinic sessions, 6 of the 98 remaining participants did not continue with home-based training: 1 experienced excessive discomfort with the AR glasses, 2 were unwilling to take Strolll home, 2 had insufficient cognitive capacity, and 1 had insufficient physical capacity. The remaining 92 participants (93.9%) took the AR glasses home for independent use. Among the 92 participants, there were 14 dropouts, 5 of whom were unrelated to the intervention, resulting in a dropout rate of 15%. Between July 2024 and February 2025, 78 out of 92 participants completed the 6-week home training with Strolll. For 5 participants, the final clinical assessment (T2) is missing due to personal circumstances, resulting in 73 participants who completed the T2 clinical assessment. Dropouts were included where available in both the feasibility and effectiveness analysis, resulting in a sample of up to 92 participants (see Suppl. Material 4).

### Clinical feasibility

#### Safety

There were no serious adverse events. Two non-injurious falls were reported while using the Strolll platform, 1 supervised in-clinic and 1 independently at home. The average weekly fall rate in daily life did not differ significantly between usual-care and intervention periods (*Z* = −0.02, *P* = .991). Twelve participants experienced dizziness at least once, 4 reported headaches, and 15 reported eyestrain (for a comprehensive overview, see Suppl. Material 6). In the Strolll evaluation questionnaire, participants generally reported feeling safe and did not express fear of falling while training at home with Strolll. Five participants disagreed with feeling safe, 2 of whom dropped out.

#### Adherence

**Figure 2:**
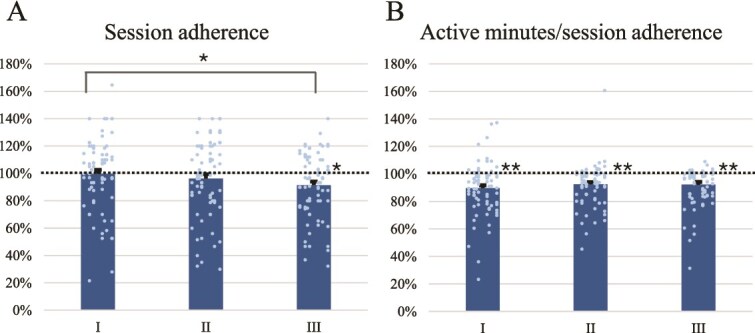
Session adherence (A) and active minutes/session adherence (B) for the first (I), second (II), and third (III) part of the Strolll intervention period at home, with the dots representing individual participants. Error bars represent the standard error of the mean. Asterisks indicate significant post hoc differences over Time or deviations from 100% (^*^P < .05 and ^**^P < .001).

Participants who completed the 6-week Strolll home training (*n* = 78) performed an average of 731 minutes (SD = 255) of active exercise. Session adherence was high, on average 96% (Figure 2A), but decreased slightly over time (*F*_2,154_ = 5.10, *P* = .007, *η_p_*^2^ = 0.062), with a significant post hoc difference between the means of the first and third parts of the intervention period (M = 99.67%, SD = 25.87 vs M = 91.38%, SD = 23.35; *t*_77_ = 3.00, *P =* .011, for which the third part deviated significantly from 100% (*Z =* 864.50, *P* = .008)). Active minutes/session adherence was 91% (Figure 2B) and did not differ over time (*F*_1.439,110.795_ = 1.69, *P* = .196, *η_p_*^2^ = 0.021), although it significantly deviated from 100% (all *Z* > 270.50, *P* < .001).

#### Game-play level and performance

**Figure 3:**
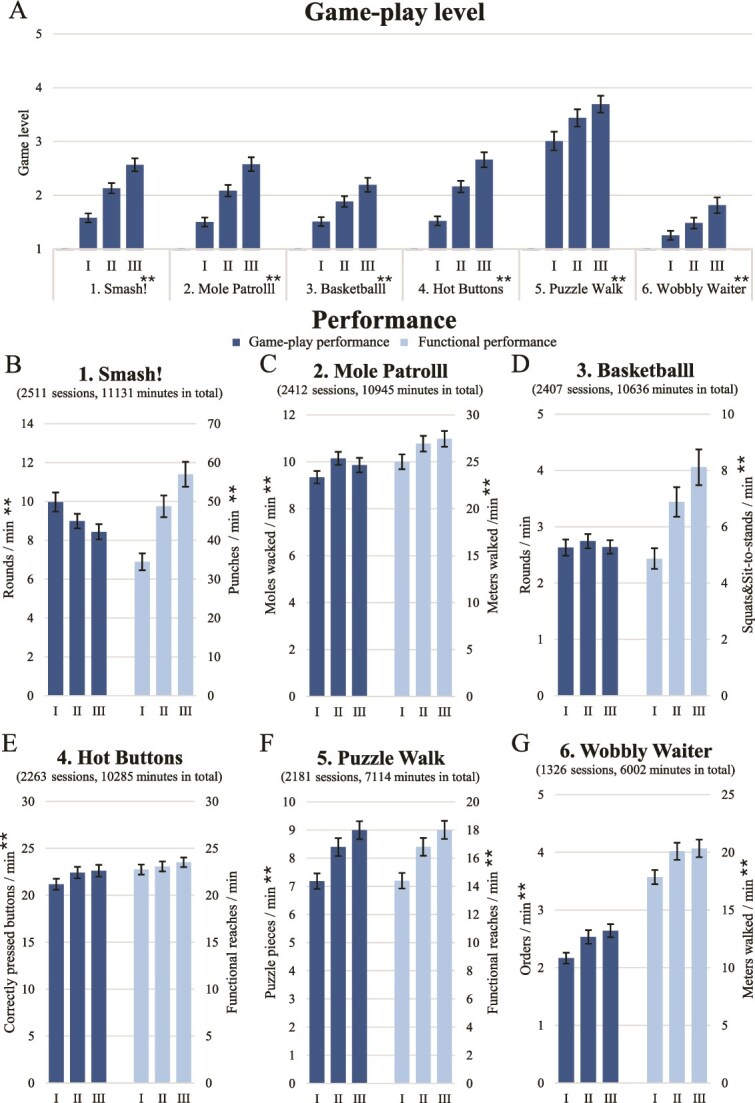
Game-play level (A), game-play performance scores (dark blue) and functional performance scores (light blue) (B-G) of gamified exercises for the first (I), second (II), and third (II) part of the Strolll intervention, ranked by frequency of play. Error bars represent the standard error of the mean. Asterisks indicate a significant main effect of Time (^**^P < .001).

The Strolll intervention was prescribed in a progressive manner, with game-play levels significantly increasing over Time for all gamified exercises (*χ*^2^_2_ > 29.94, *P* < .001), showing significant post hoc differences between all 3 intervention time points (*t* > 2.84, *P* < .05; see Figure 3A). Both game-play performance scores (eg, the number of moles wacked per minute) and functional performance scores (eg, meters walked per minute) improved significantly over time for Mole Patrolll, Puzzle Walk, and Wobbly Waiter (game-play scores: all *F* > 10.27, *P* < .001, *η_p_*^2^ > 0.119; functional scores: all *F* > 14.81, *P* < .001, *η_p_*^2^ > 0.252, see Figure 3). For Smash!, the significant decline in the game-play performance score (fewer rounds per minute; *F*_1.374,105.797_ = 9.76, *P* < .001, *η_p_*^2^ = 0.113) was accompanied by an increase in the functional performance score (more functional reaches per minute; *F*_1.304,100.405_ = 85.32, *P* < .001, *η_p_*^2^ = 0.526). For Basketballl, the game-play performance score (rounds per minute) did not change over Time (*F*_1.333,97.277_ = 1.32, *P* = .264, *η_p_*^2^ = 0.018), whereas the functional performance score (number of squats or sit-to-stands per minute) increased significantly over Time (*F*_1.220,89.046_ = 52.04, *P* < .001, *η_p_*^2^ = 0.416). These deviating findings for Smash! and Basketballl followed directly from particular gamified-exercise mechanics, where higher game-play levels required substantially more functional reaches or squats/sit-to-stands to complete a round. In Hot Buttons, the game-play performance score (correctly pressed buttons per minute) improved significantly over time (*F*_1.764,135.859_ = 10.29, *P* < .001, *η_p_*^2^ = 0.118) while the functional performance score (number of functional reaches per minute) did not vary over time (*F*_1.635,125.926_ = 1.69, *P* = .198, *η_p_*^2^ = 0.021). Figure 3 ranks the gamified exercises by frequency of play. Since Cue challenge was the least prescribed exercise, performed consistently over time by only 15 participants, associated performance scores were not analyzed.

#### User experience and technology acceptance

**Figure 4:**
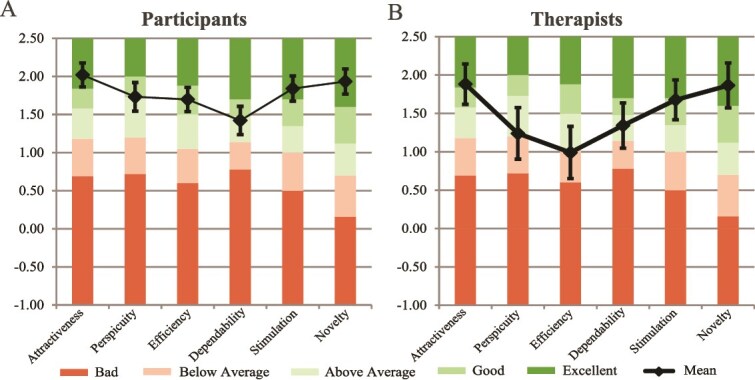
Visualization of the User Experience Questionnaire (UEQ) scores across the 6 subscales for participants (A) and therapists (B), relative to benchmark scores of the questionnaire. Error bars indicate the 95% CI.

**Figure 5:**
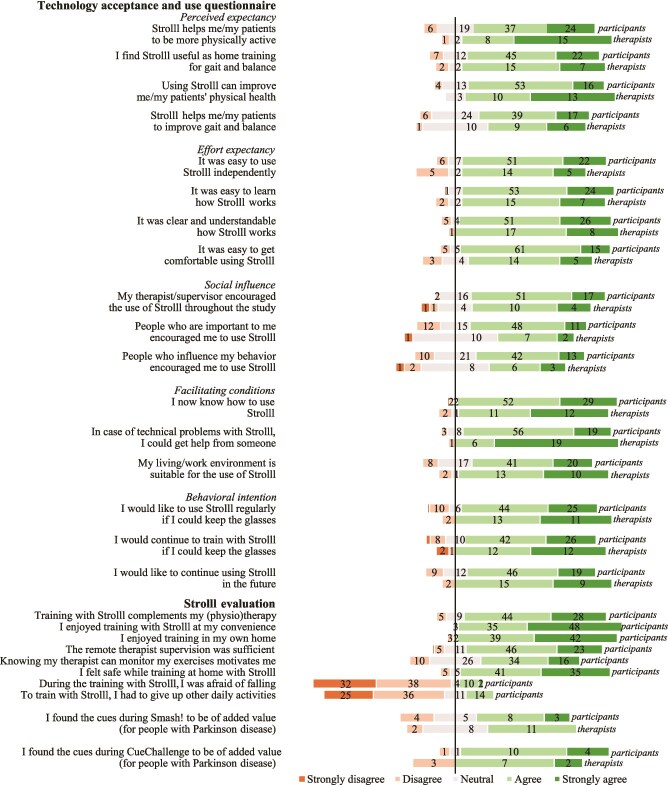
Technology acceptance and use questionnaire and the Strolll evaluation questionnaire for the 5-point Likert-scale questions.

The user experience and acceptance of the Strolll intervention were overall positive for both participants and therapists (Figures 4 and 5). All UEQ subscales were rated between “above average” and “excellent” relative to UEQ benchmarks,^22^ except for the “efficiency” subscale, which therapists rated “below average” (Figure 4B). Figure 5 shows the distribution of scores for the Likert-scale (1-5) questions of the Strolll evaluation questionnaire and the technology acceptance and use questionnaire. Across all subscales of the technology acceptance and use questionnaire, scores were generally positively rated, similarly for participants and therapists (Figure 5). That is, participants/therapists: (1) expected to/expected their patients to benefit from the intervention (perceived expectancy; participants: M = 3.90, SD = 0.63; therapists: M = 4.15, SD = 0.69), (2) experienced the technology to be easy to use (effort expectancy; participants: M = 4.08, SD = 0.58; therapists: M = 3.95, SD = 0.71), and (3) felt that significant others believe that the technology should be used (social influence; participants: M = 3.77, SD = 0.63; therapists: M = 3.52, SD = 0.89). The subscale “facilitating conditions” cannot be interpreted because of a low internal consistency for the participants (Cronbach α = .37) and should therefore be considered at item level (see Figure 5). Finally, there was a positive behavioral intention toward the technology (behavioral intention; participants: M = 3.94, SD = 0.87; therapists: M = 4.24, SD = 0.83).

The top 3 advantages of Strolll for clinical implementation identified by the participants were: (1) the ability to train at home, (2) its ability to promote increased physical activity, and (3) the enjoyable nature of the training. Therapists identified the top 3 advantages, consistent before and after delivering the Strolll intervention, as: (1) the enjoyable nature of the training, (2) its ability to promote increased physical activity, and (3) the opportunity to monitor their patients remotely. The main barriers that participants reported were: (1) technical issues (see Suppl. Material 7), (2) the need for sufficient space at home, and (3) limited variation in exercises. Before the intervention, the top 3 disadvantages reported by therapists were: (1) technical issues (see Suppl. Material 7), (2) reduced supervision, and in shared third place (3) unsuitability for every patient with Parkinson disease and financial costs. After delivering the intervention, the reported barriers shifted to: (1) technical issues, (2) financial costs, and (3) the need for specific technical skills. These top 3 rankings were determined based on how frequently and prominently participants and therapists mentioned each factor (see Suppl. Material 8 for a comprehensive overview of reported advantages and disadvantages).

### Potential effectiveness

A significant main effect of time was observed for the LME analyses for all outcome measures (TUG, Mini-BESTest, 5TSTS, 10MWT at comfortable walking speed, and FES-I; all *P* ≤ .013), except for the 10MWT at fast walking speed, showing a borderline significant effect of time (*P* = .053), see the Table 1. For the primary outcome measure, TUG, and the 10MWT at fast walking speed, only the second reverse Helmert contrast (T2-mean [T1, T0]) was significant (*t*_165.006_ = −2.39, *P* = .018 and *t*_163.992_ = −2.36, *P* = .020, respectively), indicating that improvements occurred only after the intervention. For the secondary outcome measures Mini-BESTest, 5TSTS, and 10MWT at comfortable walking speed, significant improvements were observed after both the first (T1-T0) and second reverse Helmert contrasts (T2-mean [T1, T0]); *P* ≤ .04), indicating performance improvements after usual care, with further improvement following the intervention. Regarding the FES-I, only the first reverse Helmert contrast reached significance (*t*_164.450_ = 4.23, *P* < .001), showing higher scores after the usual-care period compared to baseline, suggesting an increased concern about falling following usual care only.

**Table 1:** Main effects of time from the linear mixed-effects analyses with their reverse Helmert contrasts for the potential effectiveness concerning clinical test outcomes

Clinical test	T0	T1	T2	Main effect of time		First reverse Helmert contrast			Second reverse Helmert contrast		
	EMM (SE)	EMM (SE)	EMM (SE)	*F*(df)	*P*	*t*(df)	*P*	ΔT1-T0 (SE)	*t*(df)	*P*	ΔT2-mean (T1, T0) (SE)
TUG (s)	8.56 (0.22)	8.30 (0.22)	8.09 (0.23)	*F*_2,163.59_ = 4.45	.013^a^	*t*_162.188_ = −1.80	.075	−0.27 (0.15)	*t*_165.006_ = −2.39	.018^a^	−0.34 (0.14)
5TSTS (s)	13.79 (0.35)	12.73 (0.35)	11.99 (0.36)	*F*_2,164.09_ = 21.87	<.001^a^	*t*_162.431_ = −4.15	<.001^a^	−1.06 (0.26)	*t*_165.763_ = −5.17	<.001^a^	−1.27 (0.25)
10MWT comf (s)	8.23 (0.17)	7.99 (0.17)	7.74 (0.18)	*F*_2,163.27_ = 7.53	<.001^a^	*t*_161.885_ = −2.07	.040^a^	−0.24 (0.12)	*t*_164.664_ = −3.29	.001^a^	−0.36 (0.11)
10MWT fast (s)	6.42 (0.16)	6.36 (0.16)	6.18 (0.16)	*F*_2,163.00_ = 2.99	.053	*t*_162.003_ = −0.66	.513	−0.06 (0.09)	*t*_163.992_ = −2.36	.020^a^	−0.20 (0.09)
Mini-BESTest	23.09 (0.30)	23.80 (0.30)	24.41 (0.32)	*F*_2,164.56_ = 13.17	<.001^a^	*t*_162.418_ = 3.01	.003^a^	0.72 (0.24)	*t*_166.714_ = 4.16	<.001^a^	0.96 (0.23)
FES-I	24.20 (0.78)	26.67 (0.78)	25.70 (0.80)	*F*_2,165.44_ = 9.02	<.001^a^	*t*_164.450_ = 4.23	<.001^a^	2.47 (0.59)	*t*_166.437_ = 0.49	.625	0.27 (0.54)

a Statistically significant.

Abbreviations: 5TSTS, 5 Times Sit-to-Stand; 10MWT, 10-m walk test; comf, comfortable speed; EMM, estimated marginal means; FES-I, Falls Efficacy Scale-International; fast, fast speed; Mini-BESTest, Mini Balance Evaluation Systems Test; SE, standard error; TUG, Timed “Up & Go” test.

### AR cues

AR cues were deemed beneficial for 53 out of 92 participants. Participant characteristics (Hoehn and Yahr, Freezers, MoCA score [cognitive dysfunctions], and baseline TUG) did not differ between participants for whom AR cueing was and was not deemed beneficial (all *P* > .05). The preferred type of cue varied across participants: 2D lines (*n* = 22), 3D obstacles (*n* = 15), auditory rhythm (*n* = 12), and dinosaur footprints (*n* = 4). Cue settings were personalized, resulting in a broad range of configurations (Suppl. Material 9). Mentioned reasons for the decision to add AR cues to gamified exercises were alleviating freezing of gait (*n* = 6), improving step length (*n* = 29), walking stability (*n* = 15), foot clearance (*n* = 28), and other purposes (*n* = 11), with multiple reasons possible per participant. These findings support the hypothesis that most participants were deemed to benefit from adding AR cues and that the selected type of cue and its settings varied among participants. As an example of an individualized cueing approach, we highlight 2 participants: 1 participant preferred AR lines to alleviate freezing and take big steps, with an intercue distance of 70 cm and a line width of 50 cm, while another participant chose AR obstacles with an intercue distance of 66 cm and an obstacle height of 20 cm to better lift the feet during walking. Eventually, 46 participants used personalized AR cues at home during either Cue challenge, Smash!, or both. Both participants and therapists generally found AR cues valuable in Cue challenge, while for Smash! their opinions varied (see Figure 5).

## Discussion

This pragmatic clinical trial evaluated the clinical feasibility and potential effectiveness of Strolll, an AR neurorehabilitation platform offering gamified gait-and-balance exercises for individuals with Parkinson disease, implemented in real-world clinical practice. Furthermore, the study explored the potential of integrating personalized AR cueing into AR exercises.

### Feasibility

This study was conducted under real-world conditions within clinical practice in the Netherlands, which allowed us to evaluate Strolll beyond prior research-controlled conditions.^12^ Overall, the intervention proved to be clinically feasible in terms of safety, adherence, performance, user experience, and acceptability.

A large majority of participants (93.9%) started the independent home training with Strolll, 9 of whom withdrew for intervention-related reasons. No serious adverse events were reported during the intervention period. A few participants reported symptoms associated with cyber sickness, like headache, nausea, and dizziness (see Suppl. Material 6). Some of these symptoms may also have a different origin than cyber sickness, such as exercise-induced dizziness through squatting or turning. Nevertheless, prior research indicated that symptoms associated with cyber sickness can occur in AR applications, but that their prevalence is generally lower than in virtual reality contexts,^30^^,^^31^ which seems supported by the limited reports in the current study. Although 2 participants experienced a non-injurious fall (out of >60.000 performed exercise minutes in total, 1 supervised in clinic and 1 independent at home), the incidence rate was low relative to the study size, study duration, and nature of the target population.^32^ Furthermore, participants generally did not report fear of falling. This perception supports the notion that the trajectory from in-clinic sessions to independent home-based exercise with Strolll, under therapist prescription, is safe.

Therapists were able to prescribe, evaluate, and adjust the exercise program of the participants, based on shared decision-making with the participants, throughout the intervention in terms of exercise type (ie, prescribed gamified exercises), duration (eg, for a subset of participants [*n* = 25] the prescribed active minutes was adjusted to align with their physical capacity), game-play level, and frequency. This individualized approach presumably contributed to a high adherence compared to similar interventions targeting individuals with Parkinson disease,^33^ which was consistent with findings from our previous research-led controlled study.^12^ This highlights the importance of individually tailoring prescribed treatment programs in promoting long-term engagement and adherence and supports its feasibility when implemented in the clinical pathway.

In terms of performance, Strolll was expected to be prescribed as a progressive but achievable training. Participants demonstrated improvements over time, not only by executing higher game-play levels but also by achieving higher game-play and functional performance scores. Notably, where our prior research focused primarily on movement quantity scores (eg, number of functional reaches and meters walked),^12^ performance scores were refined by tempo metrics (eg, number of functional reaches per minute and meters walked per minute; see Figure 3 and Suppl. Material 2). As a result, we observed improvements not only in game-play performance scores (eg, number of moles wacked per minute and number of puzzle pieces placed per minute), but also in functional performance metrics (eg, number of functional reaches and meters walked per minute). These findings confirm the intended progressive nature of Strolll, also when implemented in the clinical pathway.

The overall user experience was positive among both participants and therapists. UEQ results revealed that across domains, Strolll was evaluated higher than the benchmarks, showing excellent ratings for being able to offer a novel, attractive, and stimulating technology. A notable exception was the (borderline) below-average rating for efficiency by therapists (see Figure 4), which may be due to being relatively inexperienced in using this novel advanced form of rehabilitation technology and technical inefficiencies (eg, within the web portal for managing programs). The technology acceptance and use questionnaire indicated a positive behavioral intention towards the use of Strolll, with the participants and therapists demonstrating similar ratings across most subscales. Participants described the training as enjoyable and appreciated the flexibility of being able to train at home and at their own convenience, which is in line with our previous qualitative study.^34^ Therapists particularly valued the ability to monitor their patients’ progress remotely. Reported disadvantages included technical issues, which are not uncommon in studies involving innovative digital interventions,^35^^,^^36^ limited training space at home (which hindered the use of Wobbly Waiter and Cue challenge), and perceived lack of variation in exercises. These insights were shared with Strolll Limited for future optimization of the platform.

### Potential effectiveness

The primary outcome measure, TUG, and the 10MWT at fast speed showed significant improvements after the Strolll intervention. The other secondary outcome measures, including the Mini-BESTest, 5TSTS, and 10MWT at comfortable walking speed, showed significant improvements after usual care, which limited the identification of subsequent intervention effects. Nevertheless, significant improvements in those secondary clinical outcome measures were still observed after the Strolll intervention. Although significant improvements in both primary and secondary outcome measures following the intervention did not reach the minimal clinically important difference thresholds^37–40^ (ie, no clinically meaningful effect), they were consistently observed in the expected positive direction, suggesting the potential effectiveness of Strolll in enhancing gait, balance, and fall-risk indicators. Thus, although prior improvements after usual care may have attenuated effects, these improvements were maintained and further enhanced, highlighting the potential added value of Strolll. Additionally, participants and therapists also rated the perceived expectancy for improving gait and balance positively (see Figure 5).

To conclusively validate the effectiveness of Strolll, a randomized controlled trial on clinical outcomes is warranted^41^ and initiated.^42^ Additionally, the effectiveness findings raise concerns about the sensitivity of sparsely sampled clinical outcome measures (T0, T1, and T2), due to ceiling or floor effects in relatively high-functioning groups (eg, M = 8.53, SD = 2.33 s of TUG at baseline), and symptom fluctuations common in Parkinson disease. In that sense, the observed significant improvements in more frequently sampled game-play and functional performance scores, such as increased walking distance per minute, a higher number of sit-to-stands/squats per minute, and more functional reaches per minute (Figure 3), suggest that Strolll targets aspects of gait and balance in a task-specific manner. However, similar to most of the standard clinical outcome measures, these game-play and functional performance scores primarily reflect aspects of movement quantity and tempo, while aspects of movement quality remain less explored. The rich data captured by AR glasses offers a promising opportunity to align game-play performance, functional performance, and movement-quality outcome measures more closely with the task-specific rehabilitation goals of the gamified exercises, tentatively offering a greater responsiveness to change than standard clinical assessments. Prior work has already demonstrated the utility of AR data for valid and reliable parameterization of various gait and balance aspects during standardized tests.^43–46^ Accordingly, the potential of remotely collected gait-and-balance data during at-home training will be further explored using the rich AR dataset of this trial (>60,000 minutes). At the same time, further optimization between the gamified-exercise mechanics and their task-specific training goals may strengthen the intended effects of the intervention, ultimately aiming to achieve an optimal alignment among gamified-exercise mechanics (eg, adding obstacles to Mole Patrolll), task-specific goals (eg, including obstacle avoidance under walking adaptability), and their functional or movement-quality outcome measures (eg, obstacle-avoidance success rate).

### Integration of AR cueing

Augmented reality cues were deemed beneficial for a slight majority of participants. Contrary to our expectations, these participants did not differ from non-users in baseline characteristics. This may be due to the study’s broader focus on various gait impairments, rather than exclusively targeting freezing of gait. These findings highlight the importance of personalized decision-making when integrating AR cueing into rehabilitation exercises, rather than focusing on predefined subgroups such as individuals with freezing of gait. Furthermore, both the type of cue and its settings varied over participants, supporting our expectation that cueing is not a one-size-fits-all solution.

Some participants who initially opted to use AR cueing ultimately did not incorporate it into their home training. This may have been due to either a lack of perceived benefits or practical barriers, such as insufficient space at home to effectively apply spatial AR cues. For Smash!, some participants reported that AR cues offered little or no added value, possibly because the exercise was already inherently goal-directed (ie, requiring participants to walk toward a pillar to punch an object from that pillar), making the cues feel redundant or even distracting. However, exercises designed specifically for cueing, like Cue Challenge and Wobbly Waiter, may hold promise for future research and clinical application for improving aspects of gait, such as increasing step length, reducing shuffling, alleviating freezing of gait, and improving gait speed in people with Parkinson disease.

### Limitations and considerations

The pragmatic nature of this study holds a potential bias regarding the potential effectiveness. Although all participating therapists were trained prior to the study, assessments were conducted by different clinicians at multiple sites, and minor deviations in clinical test execution cannot be excluded. This variability could have affected both the validity and the interrater reliability of the measurements. Nevertheless, this reflects the intended use of the platform in clinical practice, where such variability is expected. Besides that, therapists had no prior experience with the use of AR technology in clinical practice before this study. Increased familiarity with the technology may enhance their ability to prescribe, evaluate, and individualize the intervention to the participant, which may optimize clinical outcomes. In the current study, exercise prescription relied on therapists’ clinical expertise in combination with shared decision-making with participants and was potentially driven by adherence and game scores available in the online web portal, where exercise prescription and its tailoring were managed. Future implementation may benefit from more structured guidance (eg, decision trees or data-driven formats informed by baseline assessments of participants), which may support or guide individualization of exercise prescription while reducing variability between therapists in exercise prescription. Additionally, while the gait-and-balance assessments (T0, T1, and T2) were intended to be scheduled at the same time of day for each participant, this could not be ensured in this pragmatic trial and may have influenced potential effectiveness outcomes due to fluctuations in Parkinson’s medication status. Another important consideration is the heterogeneity of the participant population. Individuals differed not only in clinical baseline characteristics and activity levels (see Suppl. Material 5) but also in how Strolll was integrated into their regular therapy, always in addition to the in-clinic sessions, and possibly as a replacement for their usual conventional home exercises. Whether participants actually performed such prescribed conventional home exercises during usual care was not recorded in this study. Some participants already demonstrated relatively high baseline scores on clinical outcome measures, suggesting a more preserved functional status at the start of the intervention, while others were more impaired. This heterogeneity, in combination with possible floor and ceiling effects in the clinical outcome measures, may have influenced both the responsiveness to the intervention and the magnitude of observed effects, and advocates for further personalization of the intervention and outcome measures (eg, task-specific outcome measures capturing the quality of movement).

### Implications for AR in clinical practice

The findings of this study offer valuable insights for the future integration of AR technologies into clinical practice. This study was implemented in the clinical pathway of the Dutch health care system, where people with chronic neurological disorders, such as Parkinson disease, are entitled to long-term, all-year-round reimbursed therapy (after paying a limited out-of-pocket contribution). However, the increasing pressure on the health care system, where there are not enough health care professionals available to treat the increasing number of patients,^47^ calls for innovative solutions to ensure sustainable and accessible care. Digital therapeutic solutions, like Strolll, which enable remotely prescribed, home-based rehabilitation, align closely with the mission statement of care in the Netherlands^48^: promoting care at home, supporting independent care, and utilizing digital tools where possible. We showed that Strolll feasibly contributes to this mission, where therapists considered the majority of their patients (93.9%) eligible for independent, digitally supported therapy at home, potentially freeing up therapists’ time to focus more on patients with more complex needs (like the 6 participants in this study who were ineligible for independent training at home, for example due to cognitive impairment). Earlier qualitative research already suggested that people with Parkinson disease are open to AR therapy at home, complementary to physical therapy.^34^ This is substantiated by the current study, which reflected positive behavioral intentions toward AR therapy from both participants’ and therapists’ perspectives. Such home-based therapies are needed to alleviate the burden on the health care system while enhancing accessibility and continuity of care. Future studies should incorporate health-technology assessments and cost-effectiveness analyses to further evaluate the feasibility of Strolll into clinical practice. Additionally, alternative models for AR rehabilitation, other than the home-based model presented here, should be explored, such as independent or lightly supervised in-clinic use and small-group training.

Our findings also indicate that the intended use and users of Strolll could be broadened. Beyond its focus on gait, balance, and fall risk, the platform may play a valuable role in promoting increased physical activity through engaging, immersive home-based rehabilitation exercises performed at the user’s own convenience. Promoting increased physical activity is particularly relevant given the commonly low(er) activity levels in people with Parkinson disease,^49^ which are associated with negative health outcomes.^50^^,^^51^ Growing evidence suggests that exercise at an adequate intensity level may have disease-modifying effects by positively influencing neurobiological processes such as neural growth, inflammation, and brain connectivity,^52^ thereby highlighting the value of integrating physical activity into routine care for this population.^53^ In this context, the potential of Strolll to promote greater activity levels is noteworthy, which was also identified in the current study by participants and therapists, explicitly mentioning it in their top 3 advantages, as well as in prior qualitative research with this type of AR therapy.^34^

## Conclusions

This pragmatic clinical trial demonstrates that Strolll, when implemented in a real-world setting, is safe, adherable, progressive, user-friendly, and well-accepted by both individuals with Parkinson disease and their therapists. Strolll showed potential to improve gait, balance, and fall-risk indicators, although the observed effects did not exceed minimal clinically important differences. Findings on the integration of AR cueing highlight the importance of taking an individualized approach. Future development may position Strolll not only as a physical therapy tool for remotely managed independent gait-and-balance rehabilitation, but also as a technology-driven solution to promote physical activity and improve accessibility of care.

## Supplementary Material

PTJ_-_2025_-_0464_R2_Supplementary_Material_1_-_9_pzag012

PTJ_-_2025_-_0464_R2_Supplementary_Material_10_pzag012

PTJ_-_2025_-_0464_R2_Supplementary_Video_1_pzag012

PTJ_-_2025_-_0464_R2_Supplementary_Video_2_pzag012

## Data Availability

The authors confirm that the data supporting the findings of this study are available within the article and its Supplementary Material 10.
