# Supplementary material for: Cueing-assisted gamified augmented-reality home rehabilitation for gait and balance in people with Parkinson disease: feasibility and effectiveness in the clinical pathway
Source: Phys Ther. 2026 Feb 10;106(3):pzag012. doi: 10.1093/ptj/pzag012 (PMC13023367; doi:10.1093/ptj/pzag012)
Supplement: PTJ_-_2025_-_0464_R2_Supplementary_Material_1_-_9_pzag012 [file ptj_-_2025_-_0464_r2_supplementary_material_1_-_9_pzag012.pdf]

Supplementary Material 1. Transparent changes (TC) document

1. *Description of change:* Adjustment of the analysis approach: the original 'primary analysis' (as described in protocol paper) has been removed. As a result, only within-subject analyses were performed and randomization for the between-subject analyses was no longer conducted. The within-subject analyses, now designated as the primary analysis, were performed using Linear Mixed Effects (LME) analyses, instead of repeated-measures ANOVAs as pre-specified in the protocol paper. For transparency, the pre-planned within-subject analysis, using repeated-measures ANOVAs, is depicted in Supplementary Material 9.

    - *Rationale*:

      This change better aligns with the actual study design, which did not include a formal control group, as all participants initially received usual care prior to any intervention.

      From a statistical and methodological perspective, it is more suitable to consider the participants as their own controls, comparing outcomes before and after the intervention within the same individuals. Conducting a post-hoc randomization or performing a pooled analysis would not have added scientific or statistical value in this context. As such, we realized that the originally planned randomization process was not meaningful.

      The revised approach provides a clearer and more accurate representation of the real-world implementation within this study and evaluation of the intervention.

      In addition, we discussed our approach with a statistician, and concluded that a LME model was more suitable for our research design than the repeated-measures ANOVA. Whereas the repeated-measures ANOVA exclude participants with missing values via listwise deletion, the LME model uses all available data, accounts for the unequal distribution of dropouts across the study period, and reduces the risk of selection bias.

    - *Effect of change on study results*:

      As a result of this change, a direct controlled effectiveness analysis is no longer possible, since the study does not include a fully comparable control group. Instead, the focus shifts towards evaluating the potential effectiveness of the intervention by comparing outcomes within individuals over time.

2. *Description of change*: The name of the study design has been changed from "pragmatic randomized controlled trial" to "pragmatic clinical trial."
    - *Rationale*: This terminology better reflects the actual study design and the adjustment in analysis approach.
    - *Effect of change on study results*: None expected

Supplementary Material 2. Strolll exercises and cues

**Strolll exercises**				
	**Description of the exercise**	**Exercise settings**	**Feedback**	**Performance scores**
**Smash!**<br>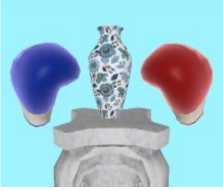	A boxing rehabilitation exercise to train gait, dynamic balance, weight shifting and turning.<br><br>The goal is to smash as many items as possible from two plinths as they appear, demanding alternate left and right punches to promote weight shifting, with available items alternating between the plinths to promote walking and turning.	• Duration of the exercise (1 - 10 minutes)<br>• Difficulty level (number of required punches before the items drop from the plinth, 2 – 20 punches)<br>• Distance between the plinths (2 - 10 meters)<br>• Optional addition of cues between plinths	*In-game feedback*<br>• Number of prescribed and performed punches<br>• Score (number of items smashed)<br><br>*Post-game feedback*<br>• Score<br><br>*Web portal feedback*<br>• Prescribed and active minutes<br>• Meters walked<br>• Number of punches<br>• Score	*Game-related performance score*<br>• Score (i.e., rounds) per minute<br><br>*Functional performance score*<br>• Functional reaches (i.e., number of punches) per minute
**Mole Patrolll**<br>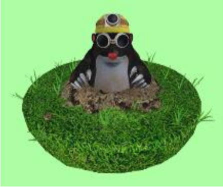	A goal-directed walking rehabilitation exercise to train gait initiation, walking adaptability, dynamic balance, turning, stopping and strength (when performed in squat mode).<br><br>The goal is to stomp as many moles as possible by scanning the room, spotting where they appear, and stomping on them either with both feet or squatting on them (a game-mode setting) before they disappear.	• Duration of the exercise (1 - 10 minutes)<br>• Difficulty level (time before mole disappears, 1 – 60 seconds per mole)<br>• Game mode (stomp or squat mode)	*In-game feedback*<br>• Score (number of moles stomped or squatted)<br>• Distance walked<br><br>*Post-game feedback*<br>• Score<br><br>*Web portal feedback*<br>• Prescribed and active minutes<br>• Meters walked<br>• Score	*Game-related performance score*<br>• Score (i.e., number of moles stomped and squatted) per minute<br><br>*Functional performance score*<br>• Meters walked per minute
**Hot Buttons**	A dynamic reaching exercise to train functional reaching,	• Duration of the exercise: (1 - 10 minutes)	*In-game feedback*<br>• Score (number of buttons pressed	*Game-related performance score*

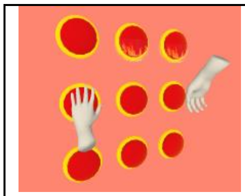	reaction time and dynamic balance.<br><br>The goal is to press the button that lights up as quickly as possible before it disappears. Feet positioning is controlled to ensure sufficient reach amplitudes.	• Difficulty level (time before button disappears, 1.5 – 60 seconds time out)<br>• Game mode (free standing, wall, table)<br>• Game mode (3, 6 or 9 buttons)<br>• Game mode (random, left hand only, right hand only)<br>• Distance (reach distance between user and board, 40 – 90 centimeters)	including bonus points for streaks which add up dependent on the number of buttons hit in a row, you lose the streak when hitting a button with the wrong hand)<br>• Number of buttons pressed in a streaks (i.e., pressing two or more buttons in a row with the prescribed hand)<br><br>*Post-game feedback*<br>• Score<br><br>*Web portal feedback*<br>• Prescribed and active minutes<br>• Number of buttons pressed<br>• Score	• Score (i.e., number of correct pressed buttons) per minute<br><br>*Functional performance score*<br>• Functional reaches (i.e., number of total buttons pressed) per minute
**Basketballl**<br>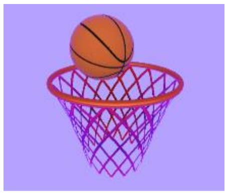	A sit-to-stand rehabilitation exercise to train dynamic balance and lower-limb muscle strength.<br><br>The goal is to score as many points as possible by completing sit-to-stand or squat-to-stand movements (a game-mode setting) to spawn a set of three basketballs, and throw them into the hoop.	• Duration of the exercise: (1 - 10 minutes)<br>• Difficulty level (number of required sit-to-stands or squats per three balls, 1 - 9 sit-to-stand/squat)<br>• Game mode (sit-to-stand, squat mode)<br>• Rhythmic music (on, off)	*In-game feedback*<br>• Number of prescribed and performed sit-to-stands or squats<br>• Number of basketballs scored<br>• Score (number of sit-to-stands or squats plus two times the number of basketballs scored)<br><br>*Post-game feedback*<br>• Score<br><br>*Web portal feedback*<br>• Prescribed and active minutes<br>• Number of sit-to-stands or squats<br>• Basketballs scored<br>• Score	*Game-related performance score*<br>• Score (i.e., rounds) per minute<br><br>*Functional performance score*<br>• Number of squats and sit-to-stands per minute

**Puzzle Walk**<br>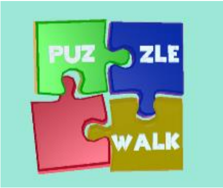 	A goal-directed walking rehabilitation exercise to train gait, dynamic balance, turning, stopping and functional reaching.<br><br>The goal is to find puzzle pieces in the room, pick them up by reaching and grabbing them with your hand and then placing them on the easel to complete the puzzle before the time runs out.	• Duration of the exercise (1 - 10 minutes)<br>• Difficulty level (number of puzzle pieces, 4 – 48 pieces)<br>• Game mode (puzzle piece height: high, hip, knee, floor)	*In-game feedback*<br>• Number of prescribed and collected puzzle pieces<br><br>*Post-game feedback*<br>• Score (number of collected puzzle pieces within the set game duration, bonus points for every second left on the clock)<br><br>*Web portal feedback*<br>• Prescribed and active minutes<br>• Meters walked<br>• Score	*Game-related performance score*<br>• Score (i.e., number of placed pieces) per minute<br><br>*Functional performance score*<br>• Functional reaches (i.e., number of collected and placed pieces) per minute
**Wobbly Waiter**<br>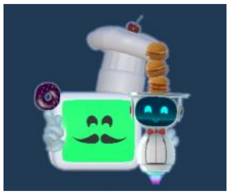	A cued rehabilitation exercise focusing on walking at a set speed, turning and standing-up/sitting-down up from/in a chair, with an element of cognitive challenge while seated (memory retention).<br><br>The goal is to memorize a cafe order from a customer, assemble the order by selecting the buttons with the correct food items in the right sequence, and deliver the order to the customer's table within a prescribed amount of time, as cued by the waiter's speed.	• Duration of the exercise (1 -10 minutes)<br>• Difficulty Level (number of items to remember, 3 – 6 items)<br>• 10-meter walk test (results)<br>• Timed Up-and-Go test (results)<br>• Gait speed adjustment (+/- 25%)	*In-game feedback*<br>• Cognitive results (number of items correctly remembered)<br>• Money collected<br>• Prescribed and performed completion durations<br><br>*Post-game feedback*<br>• Score (money collected)<br><br>*Web portal feedback*<br>• Prescribed and active minutes<br>• Meters walked<br>• Score<br>• Cognitive results<br>• completion (sub) durations	*Game-related performance score*<br>• Score (i.e., orders) per minute<br><br>*Functional performance score*<br>• Meters walked per minute
**Cue Challenge**	A cue evaluation and gait training exercise designed to try a variety of cues	• Duration of the exercise (1 - 10 minutes)	*In-game feedback*<br>• Meters walked<br>• Laps completed	NA

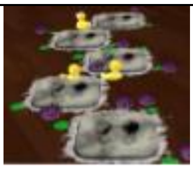	and find the one that works best for the participant. Cue Challenge comes with a multitude of settings to personalize the cues. Cue Challenge can be prescribed for gait training, modifying gait characteristics like step length and cadence with cue settings like intercue distances, along tracks that can be adjusted in terms of track shape, turning options, cue types, sizes and colors.	• Track (straight, oval, figure of eight, loop, navigation)<br>• Turning direction (left, right)<br>• Optional addition of cues to modify gait characteristics<br>• Optional Likert survey questions to record cue preference	*Post-game feedback*<br>• Score (meters walked)<br><br>*Web portal feedback*<br>• Prescribed and active minutes<br>• Meters walked<br>• Laps completed<br>• Score<br>• Answer to the Likert survey questions	

**AR cues**		
	**Description of the cue**	**Setting options**
**Lines**<br>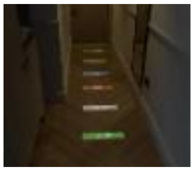	A series of flat, 2D-colored lines on the ground in front of the user to step over.	Color, step length, line width and line thickness.
**Obstacles**<br>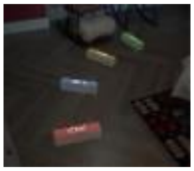	A series of 3D-colored obstacles on the ground in front of the user to step over.	Color, step length, obstacle width, obstacle height, and obstacle thickness.
**Rhythm**	An audible cue, with different action oriented rhythmic audio sounds for the user to step in time to.	Sound, volume, and speed (beats per minute).
**Dinosaur footprints**<br>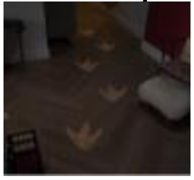	A series of dinosaur footprints on the ground in front of a user to step on with audible feedback (mud sound).	Step length and step width.

Supplementary Material 3. Main effects of Time from the pre-planned repeated-measures ANOVAs with their reverse Helmert contrasts for the potential effectiveness concerning clinical test outcomes

	T0	T1	T2	Main effect of Time			1st reverse Helmert contrast			2nd reverse Helmert contrast		
	M (SD)	M (SD)	M (SD)	*F*(df)	*p*	η_p^2	*t*(df)	*p*	ΔT1-T0 (SE)	*t*(df)	*p*	ΔT2-T1,T0 (SE)
TUG (s)	8.53 (2.33)	8.21 (1.82)	8.05 (1.73)	*F*(1.974,142.134)	**0.013**	0.059	*t*(72)= -1.986	0.051	-0.33 (0.17)	*t*(72)= -2.24	**0.028**	-0.33 (0.15)
FTSTS (s)	14.03 (3.59)	12.79 (3.44)	12.13 (3.08)	*F*(1.822,129.378)=22.35	**<0.001**	0.239	*t*(71)= -4.64	**<0.001**	-1.24 (0.27)	*t*(71)= -4.79	**<0.001**	-1.28 (0.27)
10MWT comf. (s)	8.20 (1.78)	7.86 (1.39)	7.68 (1.34)	*F*(1.530,110.165)=8.43	**0.001**	0.105	*t*(72)= -2.42	**0.018**	-0.35 (0.14)	*t*(72)= -3.52	**<0.001**	-0.36 (0.10)
10MWT fast (s)	6.38 (1.48)	6.17 (1.31)	6.08 (1.16)	*F*(1.724, 124.126)=5.17	**0.010**	0.067	*t*(72)= -2.00	**0.049**	-0.21 (0.10)	*t*(72)= -2.62	**0.011**	-0.19 (0.07)
Mini-BESTest	23.29 (3.19)	23.82 (2.93)	24.52 (2.53)	*F*(1.839,132.381)=10.78	**<0.001**	0.130	*t*(72)= 2.14	**0.036**	0.53 (0.25)	*t*(72)= 3.96	**<0.001**	0.97 (0.24)
FES-I	23.87 (6.53)	26.58 (7.55)	25.18 (7.46)	*F*(2,140)=8.16	**<0.001**	0.104	*t*(70)= 4.69	**<0.001**	2.70 (0.58)	*t*(70)= -0.07	0.948	-0.04 (0.65)

Supplementary Material 4. Flow diagram of participants in the study

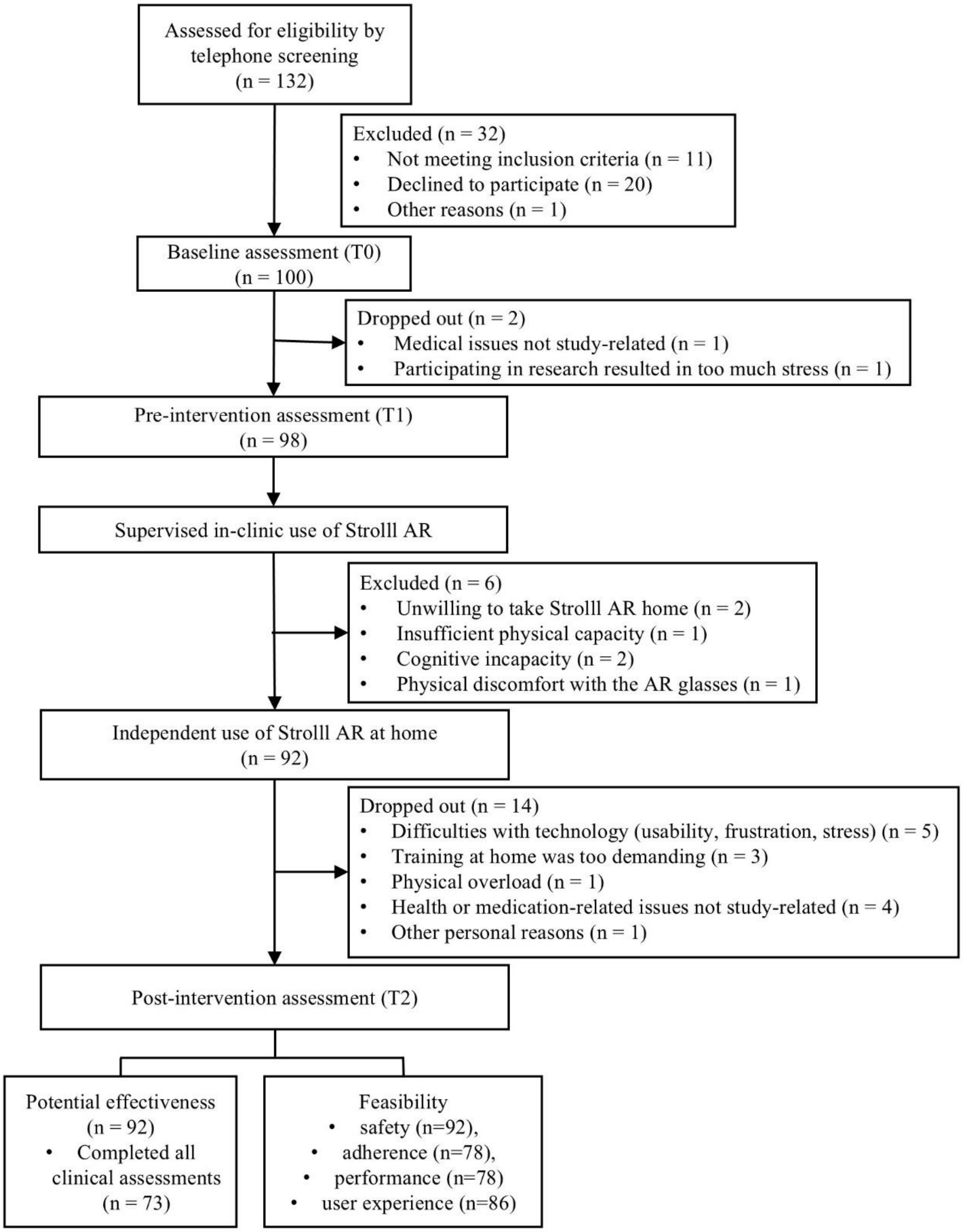

Supplementary Material 5. Clinical characteristics of participants at baseline

	N=100
**Age (years)**	69.6 ± 8.2 [42-87]
**Gender (M/F)**	72/28
**Time since diagnosis (years)**	5.4 ± 4.8 [0-25.6]
**H&Y (1/2/3)**	26/53/21
**LEDD (max mg/day)**	566 ± 355 [0-1500]
**MoCA score**	24.6 ± 3.5 [13-30]
**History of falls (past year)**	1.1 ± 1.9 [0-15]
**Freezers (yes/no)***	37/63
**MDS-UPDRS part III**	24.8 ± 10.9 [3-58]
**PASE**	126.1 ± 74.7 [0-364]

Values are presented as mean ± SD [range] if not described differently. LEDD = levodopa equivalent daily dose, H&Y = Hoehn and Yahr Scale, MoCA = Montreal Cognitive Assessment, MDS-UPDRS = MDS-Unified Parkinson's Disease Rating Scale, PASE = Physical Activity Scale for the Elderly. *Freezing is defined as a non-zero score on the New Freezing of Gait Questionnaire.

Supplementary Material 6. Reported adverse events

	Reported occurrence at home			
	**Once**	**Occasionally (< 6)**	**Frequently (>6)**	**Always**
**Serious adverse events**	0	0	0	0
**Predefined adverse events:**				
Falls	1*	0	0	0
Dizziness	7#	2	2*	0
Headache	3	0	1	0
Eyestrain	6*	4	5	0
**Other adverse events:**				
Fatigue	1*	4	0	3
Nausea	1	0	0	0
Near falls	0	1*	0	0
Stress	2^	0	0	0
Pressure on the head	0	0	0	1
Blurred vision	1	0	0	0
Back pain	0	0	1	0

	**Reported occurrence in clinic**	
	**Once**	**Twice**
Falls	1	0
Dizziness	1*	1
Nausea	0	1*
Fatigue	1*	0

Containing one (*), two (^) or three (#) dropout(s).

Supplementary Material 7. Reported technical issues

Technical issues that did and did not prevent participants from adhering to the prescribed training program (i.e., five sessions/week, 30 active minutes/session).

**Categories of issues preventing participants from adhering to the training program:**	**Number of reported issues / total number of reported issues over 6 weeks***		**Categories of issues not preventing participants to adhere to the training program:**	**Number of reported issues / total number of reported issues over 6 weeks**	
	**Reported by:**			**Reported by:**	
	**Participant**	**Therapist**		**Participant**	**Therapist**
1) The user needs to make a new room scan, but is unable to do this independently	4/30	NA	A) Handtracking issues (e.g., the user has difficulty with pushing buttons). This does not prevent the user from adhering to the program	1/31	1/14
2) The spatial map of the training area is shifted. Guiding the user to make a new scan does not solve the issue	3/30	-	B) The spatial map of the training area is shifted, requiring the user to make a new room scan	7/31	1/14
3) Due to communication issues (i.e., connection with the web portal through WiFi), games do not show in the game menu	2/30	-	D) Issues with calibrating the users' length, sitting height, arm length, resulting in misplacement of targets in games	1/31	3/14
4) Malfunctioning of hardware (e.g. AR glasses, WiFi). The user could not solve this independently	6/30	4/9	E) A game requires too many space. Changing the game setting or making a new room scan solved the problem	3/31	-
5) The user is unable to press the buttons (e.g., too far away), preventing the user from playing the games	4/30	NA	F) Games were malfunctioning (e.g., games got stuck)	5/31	5/14

6) Problems with making the room scan (e.g, the spatial mesh did not appear)	6/30	3/9	G) The game menu appeared in the wall, resulting in difficulty with pushing buttons	6/31	1/14
7) Problem with controlling the AR glasses (e.g., unable to charge the AR glasses)	3/30	NA	H) Problems due to certain light circumstances (e.g., scanning the QR-code)	5/31	-
8) A game requires too many space, preventing the user from playing the game	2/30	2/9	I) Cue was not being heared/shown	3/31	1/14
			J) Webportal issues (e.g., information is not being saved)	NA	2/14

* Technical issues reported by the therapist are issues reported over the whole clinical trial.

Supplementary Material 8. Ranked advantages and disadvantages of Strolll as reported by participants and therapists based on weighted averages with possible ties

Participants Evaluation		Therapists Expectations		Therapists Evaluation	
**Advantages**	**Disadvantages**	**Advantages**	**Disadvantages**	**Advantages**	**Disadvantages**
1. Ability to train at home	1. Technical issues	1. Enjoyable training experience	1. Technical issues	1. Enjoyable training experience	1. Technical issues
2. (Stimulating) increased physical activity	2. Required sufficient space at home	2. (Stimulating) increased physical activity	2. Reduced supervision	2. (Stimulating) increased physical activity	2. Financial costs
3. Enjoyable training experience	3. Lack of variety in exercises	3. Ability to monitor patients	3. Not suitable for every individual with Parkinson's disease	3. Ability to monitor patients	3. Required technical skills
4. Training independently at your own time	4. Technical restrictions (e.g., limited field of view)	4. User-friendly and accessible	Financial costs	4. User-friendly and accessible	4. Not suitable for every individual with Parkinson's disease
5. Improved gait and/or balance	5. Adverse events	5. Ability to train at home	5. (Time) investment for learning and set up	5. Ability to train at home	5. Reduced supervision
6. Motivational	6. High intensity	6. Practicing with cues	5. Required technical skills	6. Reduced workload for therapists	6. Lack of variety in exercises
User-friendly and accessible	7. Required self-discipline	7. Reduced workload for therapists	7. Increased fall risk without therapist supervision	7. Variety in exercises and movements to regular therapy	7. Required sufficient space at home
8. Challenging	8. Unclear scoring system	Variety in exercises and movements to regular therapy	8.Technical restrictions	Practicing with cues	8. (Time) investment for learning and set up
9. Variety in exercises and movements to regular therapy	9. Required technical skills	Improving gait and/or balance	9. High intensity	9. Training independently at patient's own time	9. Increased fall risk without therapist supervision
10. Cognitive stimulation	10. Lacking social interaction	10. Innovative approach	Required sufficient space at home	Innovative approach	10. Technical restrictions
	Difficulty fitting into daily routine				

Supplementary Material 9. Outcomes of AR cue personalization

Type of cue	Setting	Mean ± SD	Range
**2D lines (n=22)**	Step length	62.1 ± 9.1 cm	40-70 cm
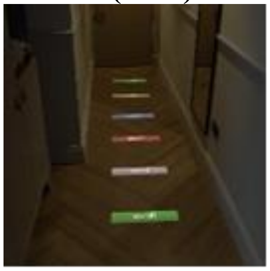	Line width	52.0 ± 5.9 cm	50-70 cm
	Line thickness	7.0 ± 3.0 cm	5-15 cm
**3D obstacles (n=15)**	Step length	70.1 ± 7.50 cm	55-80 cm
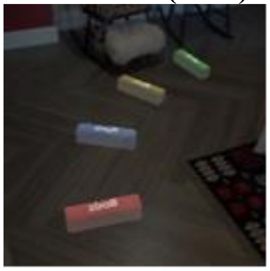	Obstacle width	50.0 ± 0.0 cm	50 cm
	Obstacle thickness	11.0 ± 2.1 cm	10-15 cm
	Obstacle height	20.7 ± 5.0 cm	10-35 cm
**Auditory rhythm (n=12)**	Pace	104.8 ± 12.4 bpm	85-127 bpm
**Dinosaur footprints (n=4)**	Step length	70.8 ± 12.9 cm	63-90 cm
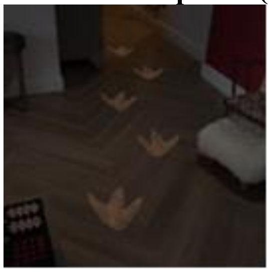	Step width	12.5 ± 5.0 cm	10-20 cm
